# Preliminary results in anterior cervical discectomy and fusion with an experimental bioabsorbable cage – clinical and radiological findings in an ovine animal model

**DOI:** 10.1186/2193-1801-2-418

**Published:** 2013-08-29

**Authors:** Dorothea Daentzer, Thilo Floerkemeier, Ivonne Bartsch, Waseem Masalha, Bastian Welke, Christof Hurschler, Theresa Kauth, Daniel Kaltbeitzel, Christian Hopmann, Bernd Kujat, Katharina Kalla

**Affiliations:** Orthopedic Department of Hannover Medical School, Diakoniekrankenhaus Annastift gGmbH, Anna-von-Borries-Str. 1-7, 30625 Hannover, Germany; Laboratory for Biomechanics and Biomaterials of Hannover Medical School, Anna-von-Borries-Str. 1-7, 30625 Hannover, Germany; Institute of Plastics Processing at RWTH Aachen, Pontstr. 49, 52062 Aachen, Germany; Institute of Materials Science of Leibniz University Hannover, An der Universität 2, 30823 Garbsen, Germany

**Keywords:** ACDF, Animal model, Bioabsorbable cage, Cervical spine, Fusion, Magnesium, Ovine animal model, Polymer, Poly-ϵ-caprolactone, Sheep

## Abstract

**Background:**

Bioabsorbable implants are not widely used in spine surgery. This study investigated the clinical and radiological findings after anterior cervical discectomy and fusion (ACDF) in an ovine animal model with an experimental bioabsorbable cage consisting of magnesium and polymer (poly-ϵ-caprolactone, PCL) in comparison to a tricortical bone graft as the gold standard procedure.

**Materials and Methods:**

24 full-grown sheep had ACDF of C3/4 and C5/6 with an experimental bioabsorbable implant (magnesium and PCL) in one level and an autologous tricortical bone graft in the second level. The sheep were divided into 4 groups (6 sheep each). After 3, 6, 12, or 24 weeks postoperatively, the cervical spines were harvested and conventional x-rays of each operated segment were conducted. The progress of interbody fusion was classified according to a three-point scoring system.

**Results:**

There were no operation related complications except for one intraoperative fracture of the anterior superior iliac spine and two cases of screw loosening and sinking, respectively. In particular, no vascular, neurologic, wound healing or infectious problems were observed. According to the time of follow-up, both interbody fusion devices showed similar behaviour with increasing intervertebral osseointegration and complete arthrodesis in 10 of 12 (83.3%) motion segments after 24 weeks.

**Conclusions:**

The bioabsorbable magnesium-PCL cage used in this experimental animal study showed clinically no signs of incompatibility such as infectious or wound healing problems. The radiographic results regarding the osseointegration are comparable between the cage and the bone graft group.

## Background

Anterior cervical discectomy and fusion (ACDF) is a standard procedure performed in patients with degenerative disc disease, disc prolapse and spinal canal stenosis. The anterior approach to the cervical spine to perform an arthrodesis was simultaneously published by Bailey and Badgley, Cloward, and Smith and Robinson (Bailey and Badgley 1960, Cloward 1958, Smith and Robinson 1958). The authors used bone dowels in cylindric- or box-shaped design, either harvested from the patient’s iliac crest or taken from human donors as allogenic material. The clinical success rate reported in literature is very high and the radiologic signs of complete bony fusion are reported to come up to 98% (Savolainen et al. 1994). There are some disadvantages of taking the bone from the iliac crest as possible complications such as infection, hematoma, fractures, and prolonged donor site pain in up to 49% of operated patients (Banwart et al. 1995). Due to the long experience with this surgical technique, this procedure is considered to be the gold standard with which all alternative therapeutic options have to be compared in regard to the clinical and radiological outcome with special interest to the fusion rate as well as to the complication rate. About 20 years ago, Kaden et al. reported the use of a titanium implant as an interbody fusion device for the cervical spine, a so-called “cage” (Kaden et al. 1993). Although the initial results are favourable, long-term effects of metallic cage devices on cervical spine motion segments are still unknown (Hacker et al. 2000, Matge 1998). Some shortcomings of metallic interbody implants, like cage migration, subsidence, adjacent level degeneration, stenotic myelopathy, and non-union have already been reported (Cabraja et al. 2012, Chen et al. 2013, Daentzer et al. 2005, Hacker et al. 2000, Majd et al. 1999, Matge 1998, Wilke et al. 2002). One further relevant disadvantage of these metallic implants is the fact, that they lead to artifacts during computed tomography (CT) and magnetic resonance imaging (MRI), complicating early detection of a metastatic recurrence and the evaluation of interbody fusion (Schulte et al. 2000). Because of their high axial compression stiffness, metallic cages may lead to stress shielding of the cancellous bone grafts inside the cage, resulting in a decreased interbody bone matrix formation or non-union (Epari et al. 2005, Kandziora et al. 2001a, 2002, Kanayama et al. 2000, van Dijk et al. 2002b). At the same time, experience with carbon fiber cages as cervical spinal interbody fusion devices had been reported (Brooke et al. 1997, Shono et al. 1993). The elasticity modulus of the carbon fiber material comes near to that of cortical bone which leads to a physiologic distribution of the forces to the adjacent endplates (Shono et al. 1993). However, subsidence is an imminent risk even during the application of carbon fiber cages (Wilke et al. 2002). Because of the radiolucent properties, carbon fiber devices do not cause any artifacts during CT or MRI. But a potential risk is the wear debris of the carbon fibers which can lead to inflammatory and foreign body reactions (Parsons et al. 1985). Since the beginning of this millennium, implants of polyetheretherketone (PEEK) have been used more frequently (Cho et al. 2002). Analogous to carbon fiber material, postoperative CT and MRI can be performed without having interfering artifacts and the evaluation of fusion is not problematic. Furthermore, the elasticity modulus of PEEK is more similar to bone than that of titanium and carbon fiber material with less risk of subsidence into the vertebral endplates. Nevertheless, subsidence in cages which consist of PEEK with consecutive segmental kyphosis had been observed in several clinical studies (Cabraja et al. 2012, Chen et al. 2013, Kast et al. 2009, Lemcke et al. 2011).

In general, the clinical success rate is supposed to be independent of the selected material (bone, titanium, carbon fiber, PEEK), because the clinical success rate seems more likely to be a consequence of the indication for the surgery and of performing a sufficient decompression in case of any stenotic pathology. However, the question of the ideal fusion cage to replace the degenerated disc is still on discussion. Bioabsorbable implants could play an important role to find the optimal material for interbody fusion, but actually they have *in vivo* only been used in animal experiments or in a few clinical studies (Chunguang et al. 2011, Kandziora et al. 2001a, b, Lippman et al. 2004, Vaccaro et al. 2004). Relevant problems seemed to be the low primary stability with development of cracks and possible foreign body reactions with inflammatory signs which raised skepticism regarding the value of bioabsorbable implants and led to a very limited use in clinical practice (Kandziora et al. 2004).

In our preclinical study we performed ACDF in an ovine model with a newly developed bioabsorbable cage as a fusion device which consisted of a magnesium structure infiltrated with a polymer (poly-ϵ-caprolactone, PCL). This material combination was chosen after *in vitro*-investigation of the mechanical properties of both substances with demonstration of an adequate initial compression strength for implantation in the cervical spine (Kauth et al. 2012). The favourable characteristics of magnesium alloys are the ability to degradation, a similar elasticity modulus to bone, a stimulation effect to bone growth and good biocompatibility (Shi et al. 2010, Staiger et al. 2006, Xu et al. 2007, Zeng et al. 2008). The PCL is known for its bioabsorbability with slow degradation, and it is radiolucent and not toxic (Albertsson and Karlsson 1995, Hiljanen-Vainio et al. 1996, Kronental 1975). The idea to combine these two kinds of material as a hybrid cage was to ensure a sufficient primary stability by the magnesium skeleton and to prevent too fast degradation by infiltration of the magnesium alloy with the PCL (Kauth et al. 2012). The goal of our investigation is to show the clinical findings, complications and the radiographic results of the harvested spinal segments (3, 6, 12, and 24 weeks after surgery) and to compare the osseointegration of the magnesium-PCL implant with the autologous bone graft.

## Materials and Methods

### Implant

**Figure 1:**
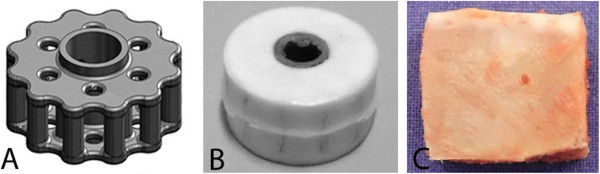
**Implants tested in the present study. A** Internal structure of the cage consisting of magnesium alloy. **B** Magnesium cage infiltrated with polymer. **C** Autologous tricortical iliac crest bone graft.

The experimental bioabsorbable implant consisted of a skeleton of the magnesium alloy AZ31 (aluminium (Al) 2.5-3.5%, zinc (Zn) 0.6-1.4%, manganese (Mn) 0.2-1.0% and silicium (Si) max. 0.3%) (Figure 1A) in a cylindric design and the absorbable polymer PCL (Boehringer Ingelheim Pharma GmbH & Co. KG, Ingelheim at Rhine, Germany) which acted as a covering to avoid too early corrosion of the magnesium structure. The inherent viscosity of the PCL was 1 dl/g, corresponding to a molecular weight of 74000 g/mol. The magnesium was first infiltrated with the PCL and in a second step, the PCL was foamed in the CESP-process (controlled expansion of saturated polymers) (Figure 1B) (Michaeli and Pfannschmidt 1999). These cages were available in different sizes (heights and depths) to optimally fill out the empty disc spaces. The autologous tricortical iliac bone grafts were harvested from the sheep and cut to a suitable form for the disc space (Figure 1C) right before the spinal surgery.

### Anaesthesia and surgical procedure

**Figure 2:**
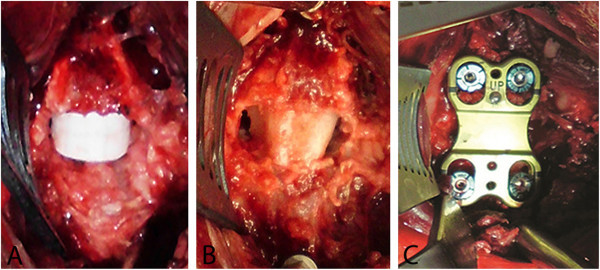
**Intraoperative images of the disc space with implants (anterior view). A** Magnesium-PCL cage. **B** Bone graft. **C** Additional stabilization with plate.

This *in vivo* investigation was approved by the State Office for Consumer Protection and Food Safety of Lower Saxony and followed the principles of laboratory animal care and all the procedures were performed in accordance with the current version of the German Law on the Protection on Animals. 24 healthy female full-grown blackcap sheep (age 2–4 years) weighing between 55.2 and 96.2 kg (mean 72.4 kg) were divided into four groups with study endpoints of 3, 6, 12, and 24 weeks after surgery. All sheep had the same operation with insertion of the bioabsorbable cage in one disc space and implantation of an autologous bone graft in a second intervertebral space with the intermediate disc space (C4/5) left intact. The distribution of the two different kinds of implants to the segments C3/4 and C5/6 was randomized, so in each group three animals had the magnesium-PCL cage in C3/4 and the autologous bone in C5/6 and three animals had the magnesium-PCL cage in C5/6 and the autologous bone in C3/4. Due to this distribution all sheep could act as their own comparison group (magnesium-PCL against bone). Anaesthesia was induced with propofol and maintained with isoflurane in oxygen. Penicillin (10 mg/kg) and carprofen (4 mg/kg) were administered directly before surgery. Penicillin injection was given on day two *post operationem* and carprofen injection was repeated with half of dose on the second and third day after surgery. The first part of the operation consisted of taking an autologous tricortical bone block from the right iliac crest. Then, a standard anterolateral approach to the cervical spine was performed. The discs C3/4 and C5/6 were completely removed while maintaining the posterior longitudinal ligament. The optimal size (height and depth) of the magnesium-PCL cage and the bone graft was defined with the help of a dummy device. The correctly dimensioned implant was centrally inserted into the empty disc space (Figure 2A, B). Finally, a titanium plate (ABC2 Anterior Cervical Plating System by Aesculap AG & Co. KG, Tuttlingen, Germany) was fixed resulting in a primary stable construction (Figure 2C). In the pilot phase, which included the operation of three sheep, a Caspar plate (Aesculap AG & Co. KG, Tuttlingen, Germany) was used. Due to implant related problems in the first two cases plates and screws were changed to the ABC2 system. The wound closure was performed in a standardized way in layers of the fascia and the subcutaneous tissue. Before the sheep recovered from anaesthesia, conventional roentgenograms of the whole cervical spine in lateral view were made with an x-ray apparatus. In the postoperative period no kind of cervical collar was used, so the mobilization was not limited. General and neurological examination took place daily during the first 10 days after surgery and every three days later on. The sheep (each six animals per group) were sacrificed after 3, 6, 12, and 24 weeks postoperatively by pentobarbital sodium overdose. Directly after euthanasia the whole cervical spines (C1-C7) were taken and then the C3/4 and C5/6 motion segments were harvested *en bloc* to perform x-rays of the 48 motion segments.

### Imaging

Immediately postoperatively, a roentgenogram of the whole cervical spine in lateral view was made. Subsequently, dependent on the time of euthanasia, the lateral x-rays were regularly repeated after 3, 6, 12, and 24 weeks to assess the implant (cage and bone graft) and to detect any hardware problems with the plate-screw-construct and to identify any gas accumulation. In order to analyse the rate of interbody fusion, only the radiographs of the monosegments C3/4 and C5/6 taken at the time of the animals’ death were encoded and reviewed in a blinded fashion according to the three-point radiographic score (RS) described by van Dijk et al. (Table 1) (van Dijk et al. 2002a, b).

**Table 1:** **Radiographic score (RS) to determine the rate of interbody fusion described by van Dijk et al. (van Dijk et al.**2002a**,**b**)**

Radiographic score	Description
RS 0	Pseudarthrosis
RS 1	Ingrowth of bone with the cage securely fixed to the vertebral bone above and below, but with a radiolucent discontinuity in the fusion mass
RS 2	Arthrodesis with solid bone bridging the fusion area

## Results

### Surgical procedure

All 24 animals recovered from the operation without unusual events. They came to normal ambulatory activities at the first day after the operation without any restrictions.

### Complications

No general intraoperative or early postoperative complications were observed, especially no neurologic or vascular problems. Furthermore, no animal had clinical indications of any infection of the wound or the deeper operation field. The first two sheep of our series operated with the Caspar plate had implant related problems. The first animal showed loosening of the upper screw pair in the lower segment after three weeks, and further had temporary signs of bronchitis. The second sheep showed relevant sinking of the cranial screws of the upper level into the disc space as well as some sinking of the cranial screws of the lower level nearly into the disc space after five weeks. Further implant related problems had not been observed. The other complications are listed in Table 2, whereas all except for the fracture of the iliac spine were not directly related to the surgical procedure. No sheep had to be excluded from the study.

**Table 2:** **Complications, time of diagnosis and consequence**

Sheep No.	Complication	Time of diagnosis after operation	Consequence
1	Temporary signs of bronchitis	2 days	Antibiotics for 10 days
	Loosening of the upper screw pair in the lower segment (C5/6, magnesium-PCL cage)	3 weeks	Euthanasia
2	Limitation in ROM of the cervical spine to one side because of pain	5 weeks	Analgesics
	Sinking of cranial screw pairs of both segments (C3/4, bone graft, and C5/6, magnesium-PCL cage)	6 weeks	Euthanasia
9	Weakness of the legs with tendency to fall after transport to outdoor area	4 weeks	Analgesics and cortisone (complete recovery)
13	Fracture of ASIS	Intraoperative	Analgesics
17	Acute massive diarrhea	3 weeks	Euthanasia

(ROM = range of motion, ASIS = anterior superior iliac spine).

### Imaging

Intraoperative fluoroscopy and directly postoperative lateral radiographs showed adequate positioning of all interbody fusion devices as well as of the plate osteosynthesis. In contrast to the Caspar plate, which showed relevant problems like screw loosening and sinking in two of three cases, the ABC2 plate worked perfectly without any signs of screw loosening or sinking or breakage at all.

**Figure 3:**
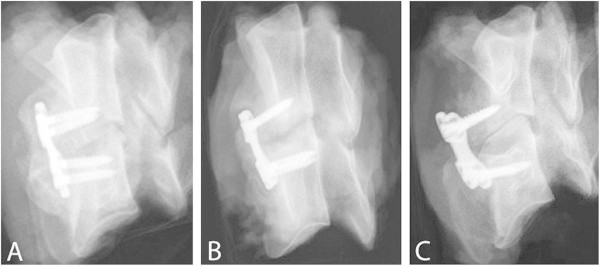
**Radiographic follow-up. A** Radiologic evidence of solid arthrodesis with complete bone bridging anterior to the motion segment and solid interbody fusion after 24 weeks, RS 2 (magnesium-PCL implant C3/4), but also with persistent evidence of the skeletal structure of the magnesium-PCL cage. **B** Radiolucent discontinuity in the interbody fusion mass and incomplete bone bridging anterior to the motion segment after 12 weeks, indicating RS 1 (bone graft C5/6). **C** No signs of interbody fusion after 6 weeks, RS 0 (bone graft C3/4).

Table 3 summarizes the results of the fusion assessments at the study endpoints after 3, 6, 12, and 24 weeks. Already after 6 weeks, most of the operated levels showed a radiographic score of 1 (RS 1) independent of the implant (magnesium-PCL cage or bone block). A similar distribution was found after 12 weeks with no motion segment demonstrating a pseudarthrosis (RS 0). After 24 weeks, 10 of 12 treated disc spaces (83.3%) showed radiologically solid bone bridging of the fusion area meaning a successful arthrodesis (RS 2). On the basis of the radiographic score, both implant types showed very similar behavior regarding the osseointegration with no obvious difference between them at all four points of follow-up. After 12 and 24 weeks, we did not find any difference between the rate of interbody fusion of both the magnesium-PCL cage and the bone graft. There were each 5/6 RS 1 and 1/6 RS 2 after 12 weeks and 1/6 RS 1 and 5/6 RS 2 after 24 weeks. It is noticeable, that after 24 weeks a remarkable bone bridging was seen anterior to all operated segments (6 with magnesium-PCL cage, 6 with iliac crest) connecting both adjacent vertebrae which indicated a complete solid fusion (Figure 3A). These findings were only inconstantly observable after 12 weeks and never visible after 3 or 6 weeks (Figure 3B, C).

**Table 3:** **Rate of interbody fusion at the time of euthanasia (after 3, 6, 12, and 24 weeks) according to three-point radiographic score (RS) of C3/4 and C5/6 motion segments (van Dijk et al.**2002a**,**b**)**

After 3 weeks (n=6)	RS 0	RS 1	RS 2
Bone graft	4	2	0
Magnesium-PCL cage	2	4	0
**After 6 weeks (n=6)**	**RS 0**	**RS 1**	**RS 2**
Bone graft	0	5	1
Magnesium-PCL cage	1	4	1
**After 12 weeks (n=6)**	**RS 0**	**RS 1**	**RS 2**
Bone graft	0	5	1
Magnesium-PCL cage	0	5	1
**After 24 weeks (n=6)**	**RS 0**	**RS 1**	**RS 2**
Bone graft	0	1	5
Magnesium-PCL cage	0	1	5

**Figure 4:**
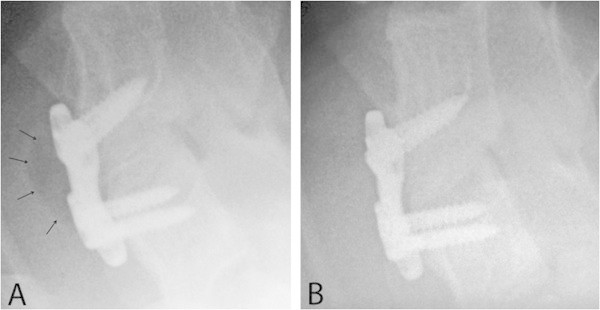
**Prevertebral gas accumulation. A** Prevertebral gas accumulation (arrows) was observed 3 weeks postoperatively in 50% of motion segments treated with the magnesium-PCL cage. **B** The same animal 6 weeks after operation demonstrating a complete disappearance of the gas within week 3 to 6 after surgery.

Another point of interest was a gas accumulation in front of the disc space which was replaced by the magnesium-PCL device, which is a known phenomenon as magnesium releases hydrogen during the corrosion process. At the lateral radiographic control after 3 weeks, a certain amount of gas collection was recognized in 50% (12 of 24 magnesium-PCL cages) of the sheep (Figure 4A). In all these cases, the gas completely disappeared within the next 3 weeks and on the following x-rays 6 weeks after operation no more gas could be seen at all (Figure 4B).

## Discussion

For evaluation of new interbody fusion devices, an appropriate animal model has to be chosen for preclinical testing. This model should be an adequate substitute to perform *in vivo* and *in vitro* studies. In search of an implant for ACDF, previous investigations have brought out the similar properties of the ovine and the human cervical spine with regard to anatomy and biomechanics (Kandziora et al. 2001a, Wilke et al. 2000, Wilke et al. 1997). In addition to the ovine cervical spine the caprine cervical spine also has been proven to be a very good model and both species were regularly used in the past for *in vivo* and *in vitro* studies to evaluate the characteristics of fusion properties as well as to analyze the biomechanical behavior of newly developed implants or substances (Chunguang et al. 2011, Kandziora et al. 2001a, Lippman et al. 2004, Kandziora et al. 2004, Cornwall et al. 2004, Goldschlager et al. 2011, Pflugmacher et al. 2004a, b, Schreiner et al. 2007, Takahashi et al. 1999, Thomas et al. 2008, Toth et al. 1995).

The main advantage of bioabsorbable materials in contrast to current cage devices (titanium, PEEK, carbon) is the potential for degradation over a distinct period of time. After that, they do not obscure postoperative radiologic assessment of intervertebral fusion and do not prevent the evaluation of the operated segment in CT or MRI because of artifacts caused by metallic implants. The optimal implant has a stiffness comparable to that of bone, which may reduce stress shielding of the graft inside the cage, possibly resulting in an accelerated interbody fusion (Chunguang et al. 2011). During the degradation process loading is transferred gradually to the healing bone and the void inside the cage is replaced with bone (van Dijk et al. 2002aSmit et al. 2003). After solid fusion of segments, cages will be absorbed, leaving no particles and avoiding great bone defects caused by removing metallic implants in revision surgery.

There are several reports about bioabsorbable implants used for ACDF in animal models (Chunguang et al. 2011, Lippman et al. 2004, Kandziora et al. 2004, Pflugmacher et al. 2004a, Thomas et al. 2008). Only few clinical studies had inserted bioabsorbable cages for ACDF in humans (Vaccaro et al. 2004). Most of these bioabsorbable cages consist of polylactide (70/30 PLDLLA, poly(L-lactide-co-D,L-lactide), which naturally degrades to carbon dioxide and water (Vaccaro et al. 2004, Kandziora et al. 2004, van Dijk et al. 2002b, Pflugmacher et al. 2004a, Smit et al. 2003). Alternative bioabsorbable materials are PCC (polymer-calciumphosphate composite), composites of 70:30 or 85:15 PLDLLA/PGA (polyglycolic acid) or MAACP/α-TCP (multiamino acid copolymer/α-tricalcium phosphate) (Chunguang et al. 2011, Lippman et al. 2004, Kandziora et al. 2004). Although most of these studies show that solid fusion can be achieved with such cages and no serious tissue response to degradation of cages is found, some negative effects have been reported (Chunguang et al. 2011, Lippman et al. 2004, Kandziora et al. 2004, Pflugmacher et al. 2004a). These were cracks with insufficient primary stability and foreign body and inflammatory reactions with consecutive osteolysis. Therefore, bioabsorbable materials have not yet reached the status of a standard implant for ACDF.

Degradable metal implants made of magnesium alloys were introduced into orthopaedic and trauma surgery in the first half of the last century and screws and plates which consisted of magnesium alloys provided stable implant materials that degraded *in vivo*, eliminating the need for a second operation for implant removal (Witte et al. 2005). To our knowledge the present study is the first one using an experimental cervical interbody fusion cage consisting of a magnesium alloy and a polymer. Therefore, the radiologic results cannot be directly compared with the results from previous studies. The radiographic analysis of the intervertebral implants had been made according to a three-point score described by van Dijk et al. to assess the grade of bony fusion (van Dijk et al. 2002a, b). After 24 weeks, almost all treated disc spaces (10 of 12, 83.3%) showed radiologically solid bone bridging of the fusion area meaning a successful arthrodesis. On the basis of the radiographic score, both implant types (magnesium-PCL cage and tricortical bone graft) showed very similar behavior and no obvious differences between both materials regarding the osseointegration at all four points of follow-up. As a result of this analysis we could assume that the magnesium-PCL implant has an identical fusion behavior and enhances osseointegration in the same manner as autologous bone, which is still the gold standard in ACDF. However, one limitation of the study is the exclusive evaluation of the fusion signs on the basis of standard lateral radiographs. In the publications of van Dijk et al. the motion segments were additionally cut to 5 mm thick parasagittal sections which also underwent lateral radiography (van Dijk et al. 2002a, b). Using these additional images the assessment of the fusion rate can be made more precisely, mainly in the inside of the cage device. Better imaging techniques to estimate the grade of interbody bony ingrowth would be analysis by CT scan or μ-CT, which makes it possible to determine the fraction of degraded implant material in detail. Furthermore, it was necessary to adapt the technique of fusion assessment described by van Dijk *et al*. to the autologous bone grafts, in which the lack of a cavity inside the bony implant has to be considered. This forms a contrast to the conditions in a “cage”, in which a fusion can actually happen by successive osseous growth through the cage (van Dijk et al. 2002a, b). However, in another report the same radiographic score was used for bone grafts and in our experience this technique proved to be appropriate also in bone grafts taken for interbody fusion because it is possible to determine the progressive building of new trabecular bone inside the bone dowel indicating increasing osseointegration (Chunguang et al. 2011).

To date, the clinical relevance of the prevertebral gas accumulation in front of the magnesium implant, which was observed after three weeks in 50% of the treated disc spaces is not clear. It is a well-known phenomenon that a certain amount of hydrogen develops as a product of magnesium corrosion during the process of degradation (Witte 2010, Witte et al. 2005, 2008). In the present study the gas constantly disappeared within week 3 to 6 after surgery, so the gas was no more seen at the 6 weeks follow-up. Clinical signs for relevant problems due to the gas like visible swelling or dysphagia did not occur.

## Conclusions

Both types of implants (magnesium-PCL cage and bone graft) showed very similar behavior on the basis of the radiographic classification, regarding the tendency to osseointegration with no obvious difference between them to all four points of time during the follow-up period and with an almost always complete arthrodesis after 24 weeks. These findings are worth to be analyzed more exactly regarding the characteristics of the experimental magnesium-PCL implant and to allow a comprehensive statement about the *in vivo* and *in vitro* behavior of the tested bioabsorbable cage by further investigations, including μ-CT as well as biomechanical and histological analysis to obtain more information regarding the degradation, the stiffness and osseointegration parameters.
